# Effects of Traditional and Bio-Based Packaging on Bioactive Compounds of Tomato By-Products During Storage

**DOI:** 10.3390/foods15071204

**Published:** 2026-04-02

**Authors:** Edmondo Messinese, Olimpia Pitirollo, Daniele Giuffrida, Francesca Rigano, Cinzia Cafarella, Roberta La Tella, Luigi Mondello, Antonella Cavazza

**Affiliations:** 1 Department of Chemistry, Life Sciences, and Environmental Sustainability, University of Parma, Parco Area Delle Scienze, 17/A, 43124 Parma, Italy; edmondo.messinese@unipr.it (E.M.); olimpia.pitirollo@unipr.it (O.P.); 2 Interdepartmental Center for Packaging (CIPACK), Parco Area Delle Scienze, 95, 43124 Parma, Italy; 3 Department of Biomedical, Dental, Morphological and Functional Imaging Sciences, University of Messina, Via Consolare Valeria, 98125 Messina, Italy; daniele.giuffrida@unime.it; 4 Messina Institute of Technology c/o Department of Chemical, Biological, Pharmaceutical and Environmental Sciences, University of Messina, Viale G. Palatucci 13, 98168 Messina, Italy; francesca.rigano@unime.it (F.R.); robertalatella@unime.it (R.L.T.); luigi.mondello@unime.it (L.M.); 5 Department of Molecular and Translational Medicine, University of Brescia, Viale Europa 11, 25123 Brescia, Italy; cinzia.cafarella@unibs.it; 6 Chromaleont S.r.l., c/o Department of Chemical, Biological, Pharmaceutical and Environmental Sciences, University of Messina, Viale G. Palatucci 13, 98168 Messina, Italy

**Keywords:** active packaging, beta carotene, biobased packaging, agro-food by-products, lycopene, storage

## Abstract

Packaging has the main role of protecting a product during storage, and the material selected for packaging has a crucial role in shelf-life control. In recent years, according to the recent European regulations on plastics, different materials have been proposed with the aim of reducing the use of fossil-based packaging. In the present work, the storage of tomato by-product powders dried at different temperatures (40 and 70 °C), in different types of packaging (plastic bag, bioplastic bag, edible active film, and edible active film enriched with antioxidants) was monitored for 11 months. Several analytical approaches were used to characterize the properties of the product after drying treatment. Oxidative stability was evaluated through the Oxitest reactor; bioactive compounds content, such as total phenolic and percentage of total antioxidant capacity, were assessed through spectrophotometric assays; high-performance liquid chromatography coupled to mass spectrometry analysis was employed for β-carotene and lycopene contents monitoring. Results showed a progressive reduction in all parameters, with slight differences in the behavior of the aliquots stored in the different materials. Samples stored in bioplastic showed a higher retention of phenolic compounds and antioxidant capacity at early storage stages, whereas conventional plastic and active packaging exhibited comparable or improved performance at later stages, depending on the analytical parameter considered.

## 1. Introduction

In the context of the Circular Economy, vegetable by-products from agro-industrial sources can be a precious source of bioactive compounds [1]. Tomato by-products (TBP), mainly peels and seeds, represent a rich source of bioactive compounds like carotenoids, lycopene, polyphenols, and antioxidants, which hold immense potential for applications in nutraceuticals, cosmetics, and functional foods [1]. However, due to the large quantities generated during the industrial processing, the stability of these compounds during storage may be critical, as light and oxygen exposure can strongly affect their bioactivity over time. In this regard, packaging materials play a key role in improving preservation, with each product requiring a tailored approach based on its unique interactions with the packaging material and the environment.

Given the substantial quantities of tomato byproducts generated during the production season, processing and utilization within a short timeframe are often impractical. Therefore, evaluating the stability of bioactive compounds during storage is essential. It is well-established that active compounds, particularly antioxidants [2,3], degrade over time due to exposure to light and oxygen, which diminishes their activity [4,5]. Identifying optimal packaging conditions is crucial to ensuring the enhanced stability of bioactive compounds over time. Packaging materials significantly influence product stability, but there is no universal solution; each product exhibits unique interactions with packaging materials and environmental factors. The selection of packaging should be treated as a specific system influenced by multiple factors, such as pH, humidity, and the product’s chemical composition, which can collectively impact the stability of both the packaging material and the product throughout its shelf life [6]. Several types of packaging materials are available for vegetables, each offering varying performances in terms of resistance, permeability, mechanical properties, and weight. Among these, plastic remains the most widely used material due to its cost-effectiveness, lightweight nature, and durability. However, from an environmental perspective, plastic poses significant ecological challenges due to its persistence in the environment post-disposal [7]. Recent EU directives [8] have emphasized the shift from conventional plastic to biodegradable materials like polylactic acid (PLA) due to the ecological concerns surrounding the environmental persistence of plastics. Bioplastics, such as PLA, offer intermediate gas barrier properties compared to materials like PET and have been shown to extend the shelf-life of fresh-cut vegetables, including tomatoes [9] and melon [10], by maintaining quality and preventing microbial contamination. However, PLA-based packaging presents limitations, including susceptibility to hydrolysis, low thermal stability, and higher cost [6,9]. Other studies on tomatoes have shown that the permeability of biodegradable films to oxygen and carbon dioxide supports proper fruit respiration, which helps prevent microbial contamination and preserves the fruit’s quality [11,12].

To address these challenges, active packaging, enriched with natural antioxidants or antimicrobial compounds, has emerged as a promising solution for enhancing the stability of stored products. For instance, active packaging formulations considered as recent green solutions are currently proposed by many researchers as an alternative to plastic materials. It generally consists of a biodegradable material enriched with antioxidant or antimicrobial compounds that can be released into the product, thereby enhancing its shelf-life over time. Many proposed examples [13,14,15,16,17] showed a great effect in prolonging shelf-life, such as in the case of fresh products like cherry tomatoes [18]. Although active and bio-based packaging materials have been extensively studied for fresh tomato products and extracts [18,19,20], comparative evaluations involving multiple packaging systems—including conventional plastics, bioplastics, and active edible films—applied to dried tomato by-product powders over extended storage periods are limited [5,19]. Existing studies on dried tomato products have primarily evaluated conventional materials over storage periods not exceeding six months and have not systematically investigated the combined effect of drying conditions and packaging type on multiple bioactive markers [19]. This study aims to investigate the effects of different drying conditions (70 °C and 40 °C) and packaging materials on the stability of dried tomato by-products (TBPs) during storage. Specifically, the effect of conventional low-density polyethylene (LDPE) packaging, a starch-based compostable bioplastic (Mater-Bi^®^), and two alginate-based edible films (with and without antioxidant enrichment) were comparatively evaluated under identical storage conditions.

Preliminary experiments included the assessment of oxidative stability using the Oxitest reactor, whereas spectrophotometric assays, namely total phenolic content (TPC) and total antioxidant capacity (%TAC), were performed after drying to characterize the initial levels of bioactive compounds. During storage, TPC and %TAC were monitored as key analytical indicators to describe time-dependent changes and to comparatively evaluate the influence of different packaging systems on the stability of bioactive compounds. In addition, the study focused on the evolution of major tomato carotenoids, namely β-carotene and lycopene, which were periodically monitored by high-performance liquid chromatography coupled with photodiode array and mass spectrometry detection. Overall, the work was designed as a comparative investigation of conventional and bio-based packaging solutions, aimed at identifying time- and material-dependent differences in the stability of bioactive compounds rather than assessing absolute preservation efficiency relative to unpackaged storage.

## 2. Materials and Methods

### 2.1. Chemicals

Water (Milli-Q), ethanol (96%), methanol (99%), DPPH (2,2-diphenyl-1-picrylhydrazyl), nylon filters (0.2 μm × 25 mm), Folin–Ciocalteu reagent, gallic acid, and PTFE (polytetrafluoroethylene) filters (0.2 μm × 25 mm) were all obtained from Agilent Technologies (Milan, Italy). Additionally, materials used for alginate film preparation, such as sodium alginate, calcium carbonate, glycerol, and D-(+)-gluconic acid-σ-lactone (GDL), were purchased from Sigma Aldrich (Steinheim, Germany). Vitamin E (Tocoblend), used as an active component for antioxidant effect, was supplied by Greci Industria Alimentare S.p.A. (Ravadese, Parma, Italy). Carotenoid standards were purchased from Extrasynthese (Genay, France). N-hexane, ethyl acetate (EtAc), and methyl tert-butyl ether (MTBE) were purchased from Merck Life Science (Darmstadt, Germany).

### 2.2. Sample Preparation, Packaging Configuration, and Storage Design

Tomato by-products (comprehending peels and seeds) were obtained from industrial processing by GRECI Industria Alimentare S.p.A. (Ravadese, Parma, Italy) during the 2023 harvest. In order to prevent fermentation, the samples were immediately dried. TBP were oven-dried at different temperatures and times to achieve the same cooking value (C = 30.9), coded as follows: TBP-70, 70 °C for 4 h; TBP-40, 40 °C for 48 h. The matrices were ground to obtain a fine powder with a homogeneous particle size. The drying efficiency was expressed as mass yield (%), while process severity was quantified using the cook value, which accounts for the combined effect of temperature and processing time.

For storage experiments, 50 g of both TBP-70 and TBP-40 were stored for 8 months at room temperature under light exposure at 23 ± 2 °C (controlled laboratory room temperature) in four different packaging types, including plastic bag (low-density polyethylene, LDPE), bioplastic bag (starch-based compostable material), alginate-based film, and alginate-based film enriched with an antioxidant. An active biobased film was prepared according to a procedure previously reported [13], using vitamin E as an antioxidant compound. For alginate-based systems, the dried tomato by-product powder was placed in direct contact with the film, which was manually wrapped around the sample to ensure full surface contact.

The packaging systems adopted in this study were designed to provide partial protection against external factors and were not intended to function as hermetically sealed barrier materials, but rather as contact-based packaging solutions. Accordingly, differences among packaging systems were mainly related to their oxygen and water vapor permeability rather than to complete barrier performance; these properties were subsequently quantified through oxygen transmission rate (OTR) and water vapor transmission rate (WVTR) measurements to support the interpretation of storage results.

For each packaging type and drying condition, samples were stored in a single package. At predefined storage times, the package was opened for analytical sampling, an aliquot of powder was withdrawn, and the remaining material was returned to storage under the same conditions until the next sampling point.

The study was designed as a comparative evaluation of conventional and bio-based packaging solutions under identical storage conditions. No unpackaged control was included in the experimental design. Given the rapid oxidation and moisture absorption of the matrices upon direct exposure to ambient air, maintaining an unpackaged reference under reproducible and hygienic conditions over an 11-month period was not considered feasible. Accordingly, the study was explicitly designed as a comparative evaluation of packaging systems under identical storage conditions. Performance assessments are expressed relatively to conventional plastic packaging, considered as reference material.

### 2.3. Oxitest Reactor Analysis

Oxidative stability was evaluated using an Oxitest reactor (Velp Scientifica, Usmate, Italy) under accelerated oxidation conditions (90 °C; 6 bar O_2_). (Velp Scientifica, Usmate, MB, Italy). The TBP-70 (2.5 g) and TBP-40 (2.5 g) were added to two different batches of sunflower oil (25 g each) and macerated in the dark for 48 h. Then, 10 g of each batch of oil was allocated to the plates of each chamber of the reactor, and the analysis was performed at 90 °C and 6 bars of oxygen. The sunflower oil was also analyzed as a positive control. The software allows for following the oxidative process and graphically displaying the curve related to the consumption of oxygen over time, thus calculating the induction period (IP).

### 2.4. Extraction Procedure

Ethanolic extractions were conducted directly after the drying treatments and at defined intervals throughout the storage period to perform spectrophotometric assays. The extraction procedure was carried out according to a previous method [21], with slight modifications. Briefly, 1 g of TBP was extracted with 35 mL of ethanol under reflux for 30 min, then through ultrasound-assisted extraction (UAE) for 15 min. After centrifugation at 6000 rpm, 20 °C for 15 min, the supernatant was separated from the solid residue, which was extracted for the second time with 35 mL of ethanol. The supernatants were unified (final volume 70 mL) and evaporated under vacuum. The extractions were performed in triplicate. The residue was resolubilized in 7 mL of ethanol, filtered with a PTFE filter 0.45 μm, and stored at −20 °C.

### 2.5. Folin-Ciocalteau Assay

Spectrophotometric analysis (Thermo Scientific™ Evolution™ 201/220, Milan, Italy) was carried out to evaluate the TPC according to the Folin–Ciocalteu (FC) method. 50 μL of the extract was mixed with 1160 μL of Milli-Q water, 300 μL of sodium carbonate 20% *w*/*w*, and 100 μL of the Folin–Ciocalteu reagent; the solution was incubated at 40 °C for 30 min. The same procedure was followed on a blank solvent. Absorbance was measured at 760 nm. The calibration curve was made using gallic acid as a standard in a range between 0.5 and 10 μg/mL in Milli-Q water, and the results were expressed as mg of gallic acid equivalents per gram of dry extract (mg GAE/g dry extract). All the analyses were performed in triplicate.

### 2.6. DPPH Assay

The protocol followed was already reported [22] with some modifications: 500 μL of the extract was added to 1.5 mL of freshly prepared 60 μM 2,2-diphenyl-1-picrylhydrazyl (DPPH) radical solution in methanol. After 30 min, the absorbance of the solution was measured at 515 nm by UV–VIS spectrophotometry (Thermo Scientific™ Evolution™ 201/220, Milan, Italy). Pure methanol was used for blank measurement. The absorbance of the blank solution was recorded at the same wavelength, but at t = 0 min. The antioxidant activity was expressed as a percentage of the scavenging capacity of the DPPH radical and calculated as follows:

(1) TAC(%) = (Ar − As)/Ar × 100

where Ar was the absorbance of the reference DPPH solution, and As was the absorbance of the sample.

### 2.7. β-Carotene and Lycopene Contents Analyses by LC-PDA-MS

An aliquot of 1 g of lyophilized sample was extracted with 10 mL of hexane, followed by 10 mL of EtAc. The mixture was sonicated for 15 min and then centrifuged for 15 min at 3000 rpm. The resulting supernatant was collected and filtered through a 0.45 µm syringe filter. This was then evaporated under vacuum until dry and then re-suspended in 1 mL of MTBE/methanol (1:1 *v*/*v*).

The HPLC instrument was equipped with a photodiode array detector (PDA) SPD-M20A, which was directly connected to the LC column outlet and serially coupled to an LCMS-2020 spectrometer (Shimadzu, Kyoto, Japan) via an atmospheric pressure chemical ionization (APCI) source for mass spectrometry (MS). Chromatographic separation was achieved on a HALO C30 column (150 mm × 4.6 mm, 2.7 mm); the mobile phases consisted of methanol/MTBE/water (90:8:2, *v*/*v*/*v*; eluent A) and methanol/MTBE/water (8:90:2, *v*/*v*/*v*; eluent B). The gradient program was applied as follows: 0 min 10% B; 35 min 95% B. The flow rate was 1 mL/min. The injection volume was 2 μL. PDA detection was applied in the 200–700 nm range with a sampling frequency of 12.5 Hz and a time constant of 0.160 s. Chromatograms were extracted at λ 450 nm. APCI-MS acquisition was performed in both negative and positive modes in the mass range 200–1200 *m*/*z* with an event time of 0.6 s, scan speed of 1875 u/s, nebulizing gas (N_2_) flow rate of 4 L/min, detector voltage of 0.5 kV, interface temperature of 300 °C, DL (desolvation line) temperature of 250 °C, heat block temperature of 300 °C, and drying gas flow of 5 L/min. The software LabSolution ver. 5.91 (Shimadzu, Duisburg, Germany) was used for data acquisition. For quantitative evaluations, calibration curves were prepared for the two standards used in the range of 0.1–200 ppm, and the analyses were carried out in triplicate.

### 2.8. Oxygen and Water Vapor Transmission Rates Analysis

The oxygen transmission rate (OTR) of the packaging materials was determined according to ASTM D3985 standard (“Standard Test Method for Oxygen Gas Transmission Rate Through Plastic Film and Sheeting Using a Coulometric Sensor”) [23]. Before analysis, all samples were preliminarily conditioned at 23 ± 2 °C and 0% relative humidity (RH). Water vapor transmission rate (WVTR) measurements were performed under controlled temperature and relative humidity conditions (38 ± 2 °C; 90 ± 5% RH) using a gravimetric permeation approach. Although test conditions were aligned with ASTM F1249 standard (‘Standard Test Method for Water Vapor Transmission Rate Through Plastic Film and Sheeting Using a Modulated Infrared Sensor’) [24], the methodology was adapted to the instrumental configuration available. These parameters were determined by using an oxygen permeability analyser (PermO_2_, ExtraSolution, Pieve Fosciana, Italy). Specifically, OTR and WVTR measurements were performed exclusively on conventional plastic (LDPE) and bioplastic materials. Due to the extremely high permeability and structural instability of alginate-based films under the selected test conditions (38 °C; 90% RH), reliable barrier measurements could not be obtained. Measurements were performed on packaging materials at the beginning of storage (T0) and after 11 months of storage (T11) to evaluate the evolution of barrier properties over time. Barrier properties were measured to support the mechanistic interpretation of storage results rather than to claim absolute compliance with standardized industrial certification procedures. Results are reported as mean ± standard deviation (*n* = 3).

### 2.9. Statistical Analysis

Collected data have been processed, and results were expressed as the mean of three different experiments ± standard deviation (SD), using Excel. Significant differences within and between groups were evaluated by the two-way analysis of variance test (ANOVA), followed by a Dunnett’s test (α = 0.05) used to compare each group with the reference (plastic bag) at different levels and Tukey’s test to determine any significant difference in the parameters selected among the investigated samples. Additional details on the two-way ANOVA applied to conventional packaging (plastic vs. bioplastic) are reported in the Supplementary Materials.

## 3. Results and Discussion

### 3.1. Evaluation of Oxidative Stability, TPC, and %TAC After Drying Treatments

A series of preliminary experiments was conducted to evaluate the influence of drying temperature on the oxidative stability, TPC, and %TAC of extracts derived from TBPs. Two drying conditions were selected before the monitoring period: a milder drying process at 40 °C (TBP-40) and a stronger drying process at 70 °C (TBP-70). Both conditions maintained a constant cook value (30.9 min), allowing a direct comparison of drying severity while maintaining comparable overall thermal load. This parameter integrates the time-temperature history of the process and quantifies the quality loss relative to an equivalent thermal treatment at 100 °C [25]. Table 1 provides details on the cook value and percentage yield after drying for each condition assessed. No significant differences were observed in drying yields, with TBP-40 yielding 39.8 ± 1.7% and TBP-70 yielding 40.4 ± 1.9% of dry weight relative to fresh weight.

The evaluation of the oxidative stability of both TBP samples after drying was performed using the Oxitest reactor. This method allows for the determination of induction period (IP) values, which measure the time required to observe a significant drop in oxygen pressure within the oxidation chamber, indicating the onset of lipid oxidation. Due to equipment limitations that require a minimum fat content of 25% for analysis, both sample matrices were added to sunflower oil, which served as a positive control. The suspensions were analyzed after a two-day infusion period.

**Figure 1:**
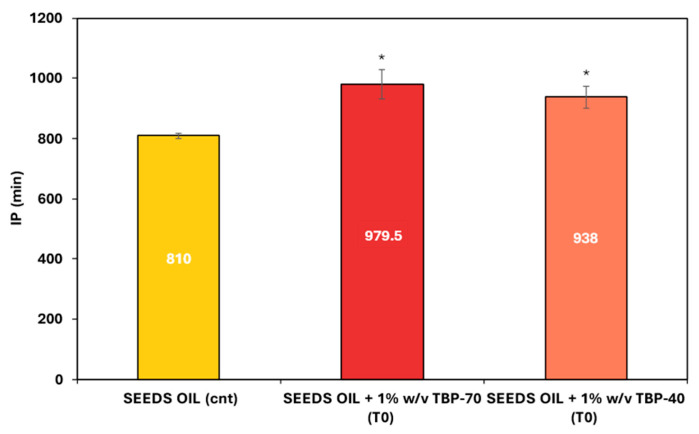
Induction period of sunflower oil before (yellow bar) and after addition of TBP-70 (red bar) and TBP-40 (orange bar). Asterisk (*) indicates statistically significant differences compared to the control.

As shown in Figure 1, the addition of TBP powders significantly enhanced the oxidative stability of sunflower oil under accelerated oxidative conditions. In both cases, supplementation of 1% (*w*/*w*) TBP resulted in a significant increase in IP values compared to the control (*p* < 0.05), confirming the antioxidant potential of the dried by-products.

In detail, the IP values of the oil enriched with TBP powders ranged from 979.5 min for TBP-70 to 938 min for TBP-40, whereas IP for untreated sunflower oil (positive control) showed significantly lower IP values, approximately 810 min. This indicates an increase of up to 40% in the induction period, thereby enhancing oxidative stability associated with the presence of antioxidant compounds [26]. These findings are consistent with previous findings, reporting that incorporating carotenoid-rich extracts (0.02% *w*/*v*) into sunflower oil significantly enhanced the induction period, as measured by the Rancimat method at 110 °C [27].

As for the effect of drying treatment on antioxidant properties, several studies have reported that high drying temperatures can positively influence oxidative stability, antioxidant capacity, and carotenoid retention. In particular, air-drying at elevated temperatures (up to 80 °C) demonstrated to increase lycopene content and antioxidant activity while reducing drying time and rate, further supporting the positive effect of higher thermal processing on antioxidant properties [28].

**Figure 2:**
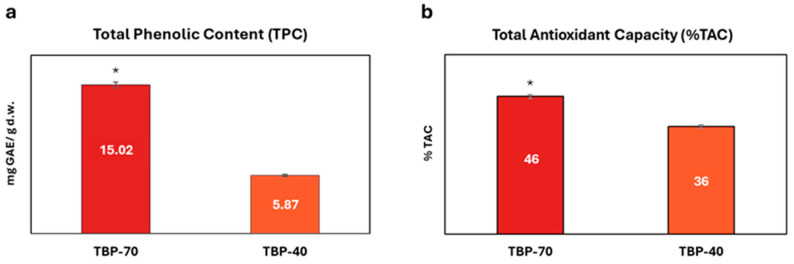
(**a**) Total Phenolic Content of TBP-70 and TBP-40 after drying treatment (T0); (**b**) total antioxidant capacity of TBP-70 and TBP-40 after drying treatment. Data are reported as mean ± SD. Statistical significance between TBP-70 and TBP-40 was evaluated by one-way ANOVA (*p* < 0.05). Asterisk (*) indicates statistically significant differences between two samples conditions.

The effect of drying temperature on TPC and %TAC is displayed in Figure 2.

As shown, marked differences in terms of TPC and %TAC were observed between the two drying conditions. TBP-70 exhibited a significantly higher TPC (15.02 ± 0.85 mg GAE/g dry weight) compared to TBP-40 (5.87 ± 0.69 mg GAE/g d.w.), corresponding to an approximately 2.5-fold increase. This increase was associated with elevated %TAC values for TBP-70 (46 ± 1%) compared to TBP-40 (36 ± 1%).

The higher TPC and %TAC values observed in TBP-70 may be partially explained by the influence of elevated drying temperatures on the release and extractability of phenolic compounds, as drying at different temperatures has been shown to alter both the profile and abundance of phenolics and their associated antioxidant activity in plant matrices [29]. Additionally, other investigations have reported that higher drying temperatures can lead to multiple heat-related transformations within the food matrix, potentially affecting the stability and measured antioxidant properties of phenolic compounds [30]. Nonetheless, the more limited increase in total antioxidant capacity (only 1.27-fold) despite total phenolic content (2.5-fold) highlights the different chemical meanings of these parameters. Indeed, while TPC reflects the overall concentration of phenolic compounds, %TAC depends on the specific structure, reactivity, and redox behavior of individual phenolics and other antioxidant components; therefore, a higher phenolic content does not necessarily translate into a proportional increase in antioxidant capacity [31].

In this regard, the contribution of thermal processes such as Maillard reactions became particularly relevant. This non-enzymatic browning reaction occurs between reducing sugars and amino acids under heat, leading to the formation of melanoidins and other bioactive compounds that contribute to the antioxidant potential of food matrices [32,33,34]. While such compounds can contribute to the overall antioxidant potential of the dried material, they may also exhibit higher chemical reactivity and reduced stability compared to native phenolic compounds. Conversely, other studies reported that higher drying temperatures can lead to a decrease in total phenol content and a subsequent reduction in carotenoid levels [35], indicating that the impact of drying temperature on antioxidant properties reflects a balance between enhanced extractability and thermally induced degradation.

### 3.2. Monitoring of TPC During Storage

Based on the differences in total phenolic content observed immediately after drying (Figure 2), the evolution of TPC during storage was monitored over a six-month period to assess the stability of phenolic compounds under different packaging conditions. Specifically, individual TPC values at each storage time for TBP-70 and TBP-40 samples are reported in Tables S1 and S2, respectively (Supplementary Material).

Extracts obtained from TBP powders stored in the four different packaging systems (plastic, bioplastic, active film, and antioxidant) were evaluated to investigate time-dependent changes in phenolic content following the application of different drying treatments.

**Figure 3:**
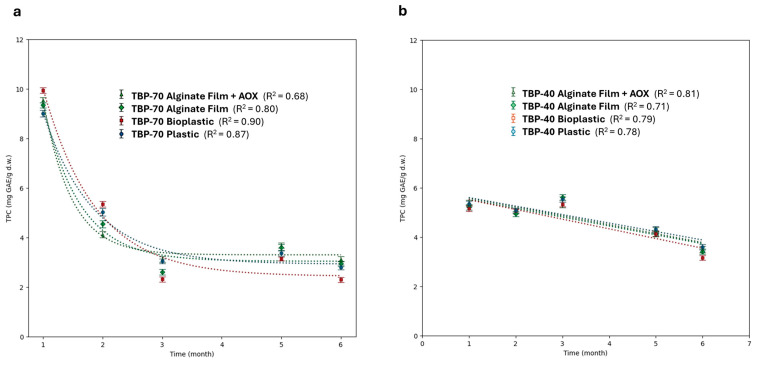
Representation of general TPC trends of (**a**) TBP-70 and (**b**) TBP-40 related to the extract obtained from TBP powders stored in different packaging materials over a six-month storage at room temperature. Statistical significance vs. plastic packaging at the same storage time was evaluated using two-way ANOVA followed by Dunnett’s test (adjusted *p*-values reported in Table S7).

As shown in Figure 3, for both TBP-70 and TBP-40 samples, storage time emerged as the dominant factor affecting TPC, as confirmed by two-way ANOVA (*p* < 0.0001). In all packaging systems, a marked decrease in TPC was observed during the early stages of storage, highlighting the intrinsic instability of phenolic compounds in dried tomato by-product powders. This general decay trend is consistent with the progressive oxidation of phenolic compounds during prolonged storage [36].

Despite this overall decreasing trend, clear differences in TPC levels and degradation kinetics were observed between the two drying treatments (see Tables S7 and S8). In particular, TBP-70 samples showed a marked decrease in TPC during the first month of storage, with values decreasing from approximately 15 mg GAE/g (T0) to around 7 mg GAE/g (T1), as shown in Figure 3a. This rapid early reduction suggests that a fraction of phenolic compounds released or generated by high-temperature drying may be more reactive or less stable and therefore more prone to oxidation or transformation during the initial storage phase [29]. In contrast, TBP-40 samples showed a more gradual decline, maintaining significantly higher TPC levels at early storage times (T1–T2) compared to TBP-70 samples (two-way ANOVA, *p* < 0.05; Table S7). This effect suggests that drying temperature plays a relevant role in determining the initial stability of phenolic compounds during storage, contributing to prolonged TPC stability over time [37,38].

Beyond these time-specific effects, the subsequent evolution of TPC suggests that packaging materials modulate phenolic stability differently depending on both drying temperature and storage duration. For TBP-70 samples, bioplastic packaging was associated with statistically higher TPC retention at selected early storage times (T1–T2; Dunnett vs. plastic, *p* < 0.05), whereas conventional plastic showed significantly higher TPC values at later storage stages (T3–T6) (see Table S7, Supplementary Material). This time-dependent behavior may depend on the different oxygen and water vapor transmission properties of the packaging materials. As reported in Table S13, bioplastic exhibited substantially higher OTR and WVTR values compared to conventional plastic, which may allow a transient protective effect at early storage stages but progressively increase oxygen exposure during prolonged storage. This behavior aligns with the intermediate gas barrier properties of biodegradable polyester-based materials, which have been reported to exhibit effective barrier performances, thereby influencing oxygen availability during storage [9,39].

Alginate-based films displayed an opposite trend, with lower TPC values compared to plastic at early storage stages but comparable values at intermediate times and significantly higher TPC retention at the end of the storage period, particularly when enriched with antioxidants (two-way ANOVA; Dunnett vs. plastic, *p* < 0.05). This trend may reflect the combined effect of oxygen permeability and antioxidant release mechanisms associated with alginate-based materials. Increased oxygen exposure at early storage stages may promote phenolic degradation, whereas at later stages, the presence of antioxidant-enriched matrices may partially counteract oxidation processes, resulting in higher TPC retention (see Table S7). In general, the observed TPC degradation patterns reflect the combined influence of drying temperature and packaging material. Drying temperature primarily governed the initial phenolic content and early degradation kinetics, whereas packaging played a more relevant role in modulating phenolic stability during prolonged storage. This interpretation is supported by the significant interaction between storage time and packaging (two-way ANOVA, *p* < 0.0001), underscoring the importance of jointly optimizing drying and packaging strategies to preserve phenolic compounds in tomato by-product powders.

### 3.3. Monitoring of %TAC During Storage

**Figure 4:**
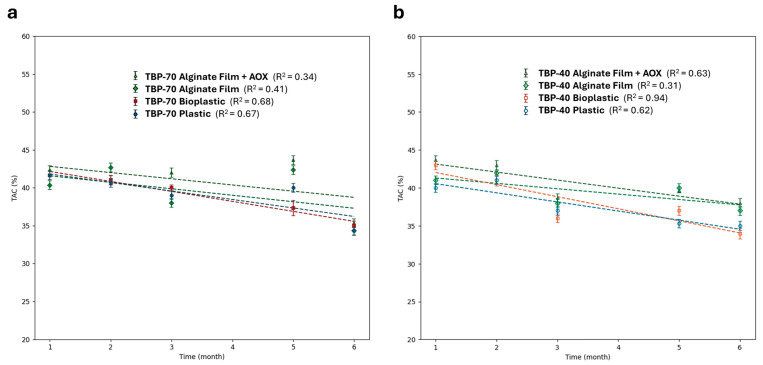
Representation of general %TAC trends of (**a**) TBP-70 and (**b**) TBP-40 related to the extract obtained from TBP powders stored in different packaging materials over a six-month storage at room temperature. Statistical significance vs. plastic packaging at the same storage time was evaluated using two-way ANOVA followed by Dunnett’s test (adjusted *p*-values reported in Table S8).

The total antioxidant capacity of TBP extracts dried and stored under different conditions is reported in Figure 4. Detailed %TAC values for TBP-70 and TBP-40 at each storage time are reported in Tables S3 and S4, respectively (Supplementary Material).

As shown, %TAC values provided a general decrease over storage time for all samples, likely due to the oxidation process. Both TBP-70 and TBP-40 exhibited statistically significant variations in %TAC depending on drying conditions and packaging type.

As shown in Figure 4a, %TAC values related to TBP-70 ranged from approximately 42% to 34%, with a pronounced decrease in the early stages (T1–T3), particularly for samples stored in conventional packaging (i.e., plastic and bioplastic). This early decline highlights a reduced stability of antioxidant capacity in TBP-70 during the initial storage phase. In contrast, active films, especially those enriched with antioxidants, maintained higher %TAC values at specific time points compared to plastic packaging (*p* < 0.05, Dunnett’s test), although a general decreasing trend was still evident. This improved performance may be attributed to the gradual release of active compounds from bio-based films, as supported by previous studies [13]. Nevertheless, %TAC also declined over time in active packaging systems, likely due to the biodegradable nature of the film and the depletion of antioxidants during storage. For TBP-40 (Figure 4b), a similar decreasing trend in %TAC was noted, ranging from approximately 40% to 32% over six months. However, the decline was less pronounced than in TBP-70. Moreover, the impact of packaging material was less evident, as plastic and bioplastic films exhibited comparable behavior, suggesting a reduced sensitivity of TBP-40 to packaging conditions, likely due to the milder drying process.

Drying conditions significantly influenced both the initial phenolic content and antioxidant capacity of TBP powders. Although TBP-70 displayed higher TPC, the corresponding increase appeared proportionally lower. This discrepancy may be related to the increased oxygen permeability in certain packaging (Table S13), accelerating oxidative degradation despite the elevated phenolic content. These findings are consistent in the literature, indicating that the correlation between total phenolic content and antioxidant capacity is not strictly linear, as it depends on phenolic composition, extraction conditions, and compound-specific reactivity [31].

Moreover, high-temperature drying can affect both the extractability and stability of phenolic compounds, potentially altering their antioxidant function. Previous studies have shown that elevated drying temperatures can cause thermal degradation or transformation of phenolics, impacting their activity during storage [30]. This explains the sharper early decrease in %TAC observed for TBP-70 compared to TBP-40.

In summary, these results underscore the importance of selecting appropriate drying protocols and packaging strategies to preserve bioactive compounds in agro-food by-products. Active films enriched with natural antioxidants partially mitigated the loss of %TAC during storage, although they did not entirely prevent antioxidant degradation. These findings align with previous research showing that PLA-based biodegradable films offer moderate oxygen barrier properties and can enhance the retention of functional compounds [9,39]. Therefore, the incorporation of antioxidants into bio-based packaging materials represents a promising strategy to reduce oxidative degradation and maintain the nutritional and functional quality of TBP powders during extended storage [16].

### 3.4. Monitoring of β-Carotene and Lycopene Contents During Storage

The contents of β-carotene and lycopene in the tomato by-product powders were monitored through high-performance liquid chromatography coupled with photodiode array and mass spectrometry detectors (LC-PDA-MS). Individual β-carotene concentrations measured during storage under the different packaging conditions are reported in Table S5 (Supplementary Material). This analytical approach provided detailed insights into the stability of these carotenoids throughout the storage period across different packaging materials. Contents of carotenoids were evaluated after two different drying treatments producing TBP-70 and TBP-40.

**Figure 5:**
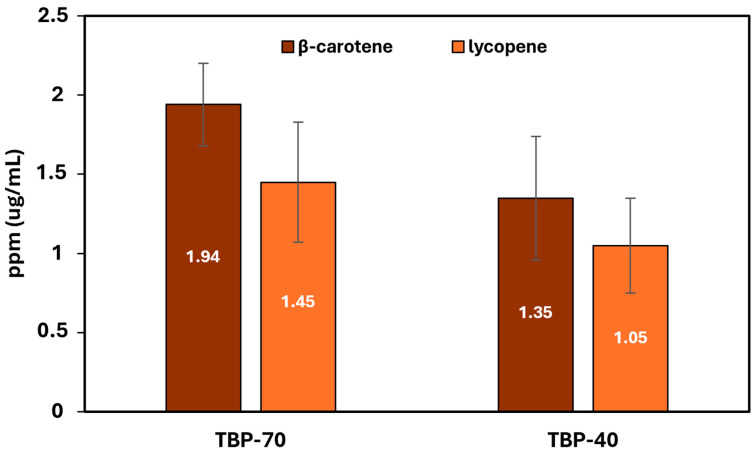
β-carotene and lycopene contents (mean ± SD) in ppm of TBP-70 and TBP-40 samples after drying.

Figure 5 displays the contents of β-carotene and lycopene immediately after drying, providing no significant differences among the two different thermal processes.

**Figure 6:**
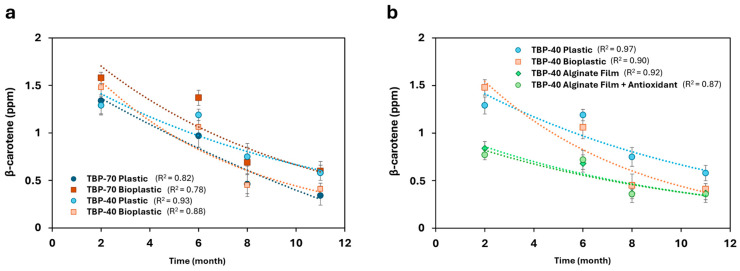
(**a**) Comparison of β-carotene contents trends (expressed in ppm) of TBP-70 stored in conventional packaging; (**b**) comparison of β-carotene contents trends of TBP-40 stored in both conventional and active packaging. Statistical significance vs. plastic packaging at the same storage time was evaluated using two-way ANOVA followed by Dunnett’s test (adjusted *p*-values reported in Tables S3 and S4).

**Figure 7:**
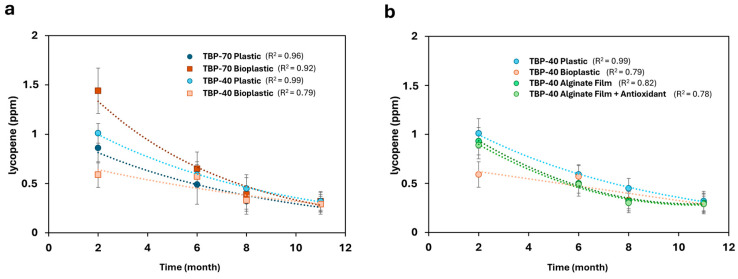
(**a**) Comparison of lycopene content trends (expressed in ppm) of TBP-70 stored in conventional packaging; (**b**) comparison of lycopene content trends of TBP-40 stored in both conventional and active packaging. Statistical significance vs. plastic packaging at the same storage time was evaluated using two-way ANOVA followed by Dunnett’s test (adjusted *p*-values reported in Tables S5 and S6).

A mixed-effects two-way ANOVA (REML) performed to specifically assess conventional packaging effects across drying treatments is reported in Table S9 (Supplementary Materials). Carotenoid monitoring started from month 2; therefore, data from the first month are not available for β-carotene and lycopene, unlike TPC and %TAC. Additionally, the comparison between packaging materials in Figure 6a and Figure 7a was limited to conventional systems (plastic and bioplastic), as alginate-based active packaging was investigated only for TBP-40 samples and therefore not included for TBP-70.

As illustrated in Figure 6 and Figure 7, both carotenoids exhibited a progressive decline in content during the 11-month monitoring period for all analyzed samples. Specifically, β-carotene content showed a marked decrease across all packaging materials over time (see Figure 6a,b), a trend that was statistically confirmed by a significant main effect of storage time in the two-way ANOVA (*p* < 0.0001). In addition, a significant interaction between storage time and packaging material (*p* < 0.05) indicated packaging-dependent degradation kinetics (Table S3).

For TBP-70 samples stored in conventional packaging, plastic exhibited the most pronounced β-carotene loss over the monitoring period (82% reduction, from 1.94 to 0.34 ppm). In contrast, bioplastic resulted in significantly higher β-carotene retention during the early storage stages (2 months; Dunnett vs. plastic, *p* < 0.05), although this protective effect diminished at later time points.

For TBP-40 samples, bioplastics exhibited a greater overall loss of β-carotene than plastic by the end of storage (72% vs. 57%), indicating reduced long-term protection under milder drying conditions. Alginate-based films, with or without antioxidants, exhibited a comparable degradation pattern, with an overall reduction of approximately 73% by the end of storage (from 1.35 to 0.37 ppm). Nonetheless, active packaging systems were investigated only for TBP-40 samples; therefore, no conclusions regarding the effect of alginate-based films on TBP-70 carotenoid stability can be drawn from this study. However, detailed Dunnett’s post hoc comparisons for TBP-40 β-carotene content at each storage time are provided in Table S10 (see Supplementary Materials).

Lycopene also exhibited a marked degradation during storage across all packaging materials and drying conditions tested (Figure 7), confirming the sensitivity of this carotenoid to storage time. Detailed lycopene concentrations at each storage time and packaging condition are reported in Table S6 (see Supplementary Material). As observed for β-carotene, storage time had a significant effect on lycopene degradation, as confirmed by the two-way ANOVA (*p* < 0.0001). Results of the mixed-effects two-way ANOVA evaluating conventional packaging across drying treatments are reported in Table S11 (Supplementary Materials).

For TBP-70 samples stored in conventional packaging, both plastic and bioplastic resulted in substantial lycopene losses, reaching approximately 80% and 77% reduction by the end of storage, respectively (Figure 7a). Bioplastic showed a less pronounced reduction at early storage stages, but this difference was no longer statistically significant at later time points, as confirmed by Dunnett’s test (*p* > 0.05), indicating convergence of degradation profiles over time (Table S5).

For TBP-40 samples, plastic and bioplastic packaging resulted in comparable lycopene degradation trends, with overall reductions of approximately 69% and 81%, respectively, by the end of storage (Figure 7b). Alginate-based films, with or without antioxidants, showed similar degradation behavior, with no statistically significant improvement in lycopene preservation compared to conventional packaging (Table S6). Accordingly, under the experimental conditions adopted in this study, alginate-based active films, even when enriched with antioxidants, exhibited a lycopene preservation performance statistically comparable to that of conventional plastic packaging, with no significant differences detected throughout the storage period.

The observed packaging-related differences at early storage stages, followed by convergence at later time points, indicate that initial barrier properties may temporarily limit oxidative degradation, whereas prolonged storage leads to degradation kinetics dominated by intrinsic carotenoid instability. Such a convergence pattern is not indicative of equivalent packaging performance but rather reflects a progressive shift in the dominant degradation mechanism. At early storage stages, differences in oxygen and water vapor barrier properties among packaging systems translated into measurable differences in bioactive retention [40]. However, as storage progressed, the intrinsic chemical instability of carotenoids—known to degrade via photo-oxidation, isomerization, and autoxidation mechanisms—became the predominant factor governing degradation kinetics [40,41], thereby attenuating the differences attributable to packaging barrier properties. This interpretation is consistent with the humidity- and time-dependent changes in gas barrier properties reported for bioplastic materials [42,43] and with the progressive increase in OTR and WVTR observed for the starch-based bioplastic in this study (Table S13).

However, the incorporation of antioxidants into alginate-based films did not significantly improve lycopene preservation, indicating that, under the conditions tested, antioxidant activity was insufficient to provide an advantage beyond that achieved with plastic packaging. The statistical evaluation of innovative packaging effects on TBP-40 lycopene content is detailed in Table S12 (Supplementary Materials).

### 3.5. Barrier Properties of Conventional Packaging Materials

The barrier properties of packaging materials play a crucial role in modulating the stability of bioactive compounds during storage, particularly for oxidation-sensitive constituents such as phenolics and carotenoids. Oxygen (OTR) and water vapor transmission rate (WVTR) measurements (see Table S13, Supplementary Materials) provided useful insights into the time-dependent performance of conventional plastic and bioplastic packaging materials and their influence on the observed degradation trends.

Bioplastic packaging exhibited substantially higher OTR and WVTR compared to conventional plastic at the beginning of storage, with a marked increase after prolonged aging. This behavior indicates a limited long-term barrier effectiveness against oxygen and moisture ingress, which may accelerate oxidation-driven degradation processes during storage. In contrast, conventional plastic showed consistently lower OTR and WVTR values and a more stable barrier performance over time, supporting its superior ability to preserve bioactive compounds during extended storage periods.

These permeability differences help explain the time-dependent effects observed in the storage experiments. The improved retention of total phenolic content and antioxidant capacity observed for bioplastic packaging at early storage stages may be attributed to transient protective effects, potentially related to initial material properties or reduced headspace oxygen exposure. However, as storage progressed, the progressive increase in oxygen and moisture permeability likely diminished this protective effect, resulting in accelerated degradation and convergence with or inferior performance compared to conventional plastic.

Similarly, the limited effectiveness of alginate-based films in preserving carotenoids can be rationalized by their barrier characteristics. Due to the highly permeable nature of alginate-based materials, reliable measurements of oxygen and water vapor transmission rates could not be obtained. Nevertheless, their intrinsic permeability to oxygen and moisture, even in the presence of antioxidants, likely constrained their ability to counteract oxidative degradation of highly sensitive compounds such as β-carotene and lycopene during prolonged storage.

In general, these findings highlight that packaging performance cannot be interpreted as a static property but rather as a time-dependent factor that interacts with the intrinsic stability of bioactive compounds and the severity of the applied drying conditions. The integration of permeability data therefore strengthens the mechanistic interpretation of storage behavior, supporting the observed trends in phenolic compounds, antioxidant capacity, and carotenoid stability.

## 4. Conclusions

This study investigated the effects of drying temperatures and packaging materials on the preservation of bioactive compounds in tomato by-products over storage, highlighting the critical role of both initial processing conditions and packaging selection in maintaining the stability of key antioxidants, including β-carotene, lycopene, and phenolic compounds. The comparison between two drying conditions, 70 °C for 4 h (TBP-70) and 40 °C for 48 h (TBP-40), showed that higher drying temperatures increased total phenolic content and total antioxidant capacity immediately after processing; however, TBP-40 showed better long-term stability, suggesting that milder drying conditions reduce degradation rates during storage.

The performance of four packaging materials—plastic (LDPE packaging), bioplastic (starch-based packaging), alginate films, and antioxidant-enriched active films—was also evaluated in terms of preservation of carotenoids such as β-carotene and lycopene contents. In the early storage phase, bioplastic showed statistically higher retention at early storage stages for TPC and %TAC compared to plastic, suggesting a moderate protective effect against oxidative degradation. However, over time, plastic provided better long-term stability while bioplastic appeared to degrade or lose its protective barrier properties. Active packaging formulations, particularly those enriched with antioxidants, showed a significant contribution to phenolic compound retention during storage, especially at later storage stages, supporting their potential as a sustainable alternative for extending the shelf-life of TBP. The stability of β-carotene and lycopene varied, with bioplastic providing better initial preservation of β-carotene, although plastic ensured more consistent retention throughout storage, while lycopene showed significant degradation under all conditions, highlighting its susceptibility to environmental factors such as oxygen and light exposure. For carotenoids, alginate-based active films showed a preservation performance comparable to conventional plastic packaging, with no statistically significant differences observed.

These findings underscore the critical roles of both drying temperatures and packaging materials in bioactive compound preservation during storage, emphasizing that while bioplastics present an eco-friendly alternative, it requires further optimization to enhance their long-term protective performances. Active packaging enriched with antioxidants represents a promising strategy for the preservation of phenolic compounds in tomato by-products powders, while providing a preservation performance comparable to conventional plastic packaging for oxygen-sensitive carotenoids under the conditions tested. This research contributes to the growing body of knowledge on food by-product valorization and sustainable packaging, aligning with the urgent need to develop materials that balance both nutritional preservation and environmental sustainability.

## Figures and Tables

**Table 1:** Drying conditions, cook values, and mass yields (%) of the two drying treatments applied to tomato by-products.

Sample	Temperature (°C)	Drying Time (h)	Cook Value * (Min)	Yield ** (%)
TBP-40	40	48	30.9	39.8 ± 1.7
TBP-70	70	4	30.9	40.4 ± 1.9

* Cook value (min) represents the cumulative thermal load applied for drying treatments, calculated according to the standard cook value approach, integrating time-temperature history into a single severity parameter. ** Yield (%) refers to the mass yield after drying, calculated as the ratio between the dry mass obtained after the drying process and the initial wet mass of the raw material, expressed as a percentage (*w*/*w*).

## Data Availability

The original contributions presented in this study are included in the article/Supplementary Materials. Further inquiries can be directed to the corresponding author.

## Supplementary Materials

The following supporting information can be downloaded at: https://www.mdpi.com/article/10.3390/foods15071204/s1. Table S1: Two-way ANOVA results for Total Phenolic Content (TPC); Table S2: Tukey’s multiple comparison test for TPC at T0 (0 weeks); Table S3: Tukey’s multiple comparison test for TPC at T2 (2 weeks); Table S4: Tukey’s multiple comparison test for TPC at T6 (6 weeks); Table S5: Tukey’s multiple comparison test for TPC at T8 (8 weeks); Table S6: Tukey’s multiple comparison test for TPC at T11 (11 weeks); Table S7: Two-way ANOVA results for Total Anthocyanin Content (TAC); Table S8: Tukey’s multiple comparison test for TAC at T0 (0 weeks); Table S9: Tukey’s multiple comparison test for TAC at T2 (2 weeks); Table S10: Tukey’s multiple comparison test for TAC at T6 (6 weeks); Table S11: Tukey’s multiple comparison test for TAC at T8 (8 weeks); Table S12: Tukey’s multiple comparison test for TAC at T11 (11 weeks); Table S13: Summary of metadata and author affiliations for supplementary data identification.
