# Supplementary material for: Effects of Traditional and Bio-Based Packaging on Bioactive Compounds of Tomato By-Products During Storage
Source: Foods. 2026 Apr 2;15(7):1204. doi: 10.3390/foods15071204 (PMC13072730; doi:10.3390/foods15071204)
Supplement: Supplementary file 1 [file foods-15-01204-s001.zip › foods-4099085-supplementary.pdf]

Supplementary Information

Effects of traditional and bio-based packaging on bioactive compounds from tomato by-products during storage

Edmondo Messinese [1,2], Olimpia Pitirollo [1], Daniele Giuffrida [3], Francesca Rigano [4], Cinzia Cafarella [5], Roberta La Tella [4], Luigi Mondello [4,6] , Antonella Cavazza* [1,2]

[1] Department of Chemistry, Life Sciences, and Environmental Sustainability, University of Parma, Parco Area delle Scienze, 17/A – 43124 Parma, Italy; edmondo.messinese@unipr.it; olimpia.pitirollo@unipr.it; antonella.cavazza@unipr.it*

[2] Interdepartmental Center for Packaging, CIPACK, Parco Area delle Scienze, 95 – 43124 Parma, Italy;

[3] Department of Biomedical, Dental, Morphological and Functional Imaging Sciences, University of Messina, Via Consolare Valeria, 98125, Messina, Italy; daniele.giuffrida@unime.it

[4] Messina Institute of Technology c/o Department of Chemical, Biological, Pharmaceutical and Environmental Sciences, former Veterinary School, University of Messina, Viale G. Palatucci snc, 98168, Messina, Italy francesca.rigano@unime.it; robertalatella@unime.it; luigi.mondello@unime.it

[5] Department of Molecular and Translational Medicine, University of Brescia, Viale Europa 11, 25123 Brescia, Italy cinzia.cafarella@unibs.it

[6] Chromaleont S.r.l., c/o Department of Chemical, Biological, Pharmaceutical and Environmental Sciences, former Veterinary School, University of Messina, Viale G. Palatucci snc, 98168, Messina, Italy

***** Correspondence: antonella.cavazza@unipr.it

Table S1. Total phenolic content (TPC, mg GAE/g dry weight) of tomato by-product powders dried at 70 °C (TBP-70) and stored for up to six months under different packaging conditions (plastic, bioplastic, alginate-based film, and alginate-based film enriched with antioxidants). Values are reported as mean ± SD (n = 3).

Storage condition	TBP-70 Plastic	TBP-70 Bioplastic	TBP-70 Alginate Film	TBP-70 Alginate Film + Antioxidant
**1**	9.01 ± 0.13	9.94 ± 0.11	9.34 ± 0.11	9.55 ± 0.10
**2**	5.04 ± 0.15	5.35 ± 0.12	4.55 ± 0.15	4.13 ± 0.13
**3**	3.05 ± 0.10	2.32 ± 0.11	2.62 ± 0.11	3.13 ± 0.11
**5**	3.37 ± 0.11	3.15 ± 0.08	3.61 ± 0.12	3.69 ± 0.10
**6**	2.81 ± 0.09	2.31 ± 0.11	2.95 ± 0.11	3.14 ± 0.10

Table S2. Total phenolic content (TPC, mg GAE/g dry weight) of tomato by-product powders dried at 40 °C (TBP-40) and stored for up to six months under different packaging conditions (plastic, bioplastic, alginate-based film, and alginate-based film enriched with antioxidants). Values are reported as mean ± SD (n = 3).

Storage condition	TBP-40 Plastic	TBP-40 Bioplastic	TBP-40 Alginate Film	TBP-40 Alginate Film + Antioxidant
**1**	5.36 ± 0.11	5.17 ± 0.11	5.22 ± 0.11	5.40 ± 0.11
**2**	5.09 ± 0.10	5.06 ± 0.08	4.95 ± 0.11	5.07 ± 0.12
**3**	5.54 ± 0.11	5.34 ± 0.10	5.63 ± 0.110	5.36 ± 0.15
**5**	4.33 ± 0.12	4.14 ± 0.11	4.14 ± 0.12	4.29 ± 0.11
**6**	3.60 ± 0.11	3.17 ± 0.10	3.42 ± 0.10	3.51 ± 0.13

Table S3. Total antioxidant capacity (%TAC) of tomato by-product powders dried at 70 °C (TBP-70) and stored for up to six months under different packaging conditions (plastic, bioplastic, alginate-based film, and alginate-based film enriched with antioxidants). Values are reported as mean ± SD (n = 3).

Storage condition	TBP-70 Plastic	TBP-70 Bioplastic	TBP-70 Alginate Film	TBP-70 Alginate Film + Antioxidant
**1**	42 ± 0.6	42 ± 0.6	40 ± 0.6	42 ± 0.6
**2**	41 ± 0.6	41 ± 0.6	43 ± 0.6	41 ± 0.6
**3**	39 ± 0.6	40 ± 0.4	38 ± 0.6	42 ± 0.6
**5**	40 ± 0.6	37 ± 1.0	42 ± 0.6	44 ± 0.6
**6**	34 ± 0.6	35 ± 0.6	34 ± 0.6	35 ± 0.6

Table S4. Total antioxidant capacity (%TAC) of tomato by-product powders dried at 40 °C (TBP-40) and stored for up to six months under different packaging conditions (plastic, bioplastic, alginate-based film, and alginate-based film enriched with antioxidants). Values are reported as mean ± SD (n = 3).

Storage condition	TBP-40 Plastic	TBP-40 Bioplastic	TBP-40 Alginate Film	TBP-40 Alginate Film + Antioxidant
**1**	36 ± 0.6	36 ± 0.6	36 ± 0.6	36 ± 0.3
**2**	40 ± 0.6	43 ± 0.6	41 ± 0.6	44 ± 0.6
**3**	41 ± 0.6	41 ± 0.7	42 ± 0.3	43 ± 0.6
**5**	37 ± 0.6	36 ± 0.6	38 ± 0.6	39 ± 0.6
**6**	35 ± 0.6	37 ± 0.6	40 ± 0.6	40 ± 0.3

Table S5. β-Carotene content (ppm) of tomato by-product powders subjected to different drying treatments (TBP-70 and TBP-40) and stored under selected packaging conditions during the storage period. Values are reported as mean ± SD (n = 3).

Storage condition	TBP-70 Plastic	TBP-70 Bioplastic	TBP-40 Plastic	TBP-40 Bioplastic	TBP-40 Alginate Film	TBP-40 Alginate Film + Antioxidant
2	1.34 ± 0.50	1.58 ± 0.60	1.29 ± 0.30	1.48 ± 0.60	0.84 ± 0.30	0.77 ± 0.10
6	0.97 ± 0.20	1.37 ± 0.50	1.19 ± 0.30	1.06 ± 0.20	0.68 ± 0.20	0.72 ± 0.10
8	0.46 ± 0.10	0.69 ± 0.20	0.75 ± 0.10	0.45 ± 0.20	0.37 ± 0.10	0.36 ± 0.10
11	0.34 ± 0.10	0.60 ± 0.10	0.58 ± 0.20	0.41 ± 0.20	0.37 ± 0.10	0.36 ± 0.10

Table S6. Lycopene content (ppm) of tomato by-product powders subjected to different drying treatments (TBP-70 and TBP-40) and stored under selected packaging conditions during the storage period. Values are reported as mean ± SD (n = 3).

Storage condition	TBP- 70 Plastic	TBP-70 Bioplastic	TBP-40 Plastic	TBP-40 Bioplastic	TBP-40 Alginate Film	TBP-40 Alginate Film + Antioxidant
2	0.86 ± 0.15	1.44 ± 0.17	1.01 ± 0.15	0.59 ± 0.13	0.93 ± 0.14	0.89 ± 0.14
6	0.49 ± 0.20	0.65 ± 0.20	0.59 ± 0.10	0.57 ± 0.11	0.50 ± 0.20	0.49 ± 0.12
8	0.32 ± 0.10	0.39 ± 0.10	0.45 ± 0.10	0.33 ± 0.10	0.32 ± 0.10	0.30 ± 0.10
11	0.29 ± 0.10	0.32 ± 0.10	0.32 ± 0.20	0.29 ± 0.10	0.30 ± 0.10	0.29 ± 0.10

Table S7. Dunnett's multiple comparisons test (vs plastic packaging) for Total Phenolic Content (TPC) at each storage time. Two-way ANOVA was performed considering *storage time* and *packaging* as factors; post hoc comparisons were carried out using Dunnett's test vs plastic at the same storage time. Data are reported as adjusted p-values (α = 0.05).

Drying temp	Storage time (months)	Comparison	Mean diff	95% CI of diff	Adjusted p value	Sig
40 °C	0	Plastic vs. Alginate Film	0	-0.2998 to 0.2998	>0.9999	ns
40 °C	0	Plastic vs. Alginate Film + Vit. E	0	-0.2998 to 0.2998	>0.9999	ns
40 °C	0	Plastic vs. Bioplastic	0	-0.2998 to 0.2998	>0.9999	ns
40 °C	1	Plastic vs. Alginate Film	0.1267	-0.05560 to 0.3089	0.1144	ns

40 °C	1	Plastic vs. Alginate Film + Vit. E	-0.01	-0.2427 to 0.2227	0.9916	ns
40 °C	1	Plastic vs. Bioplastic	0.1767	0.01817 to 0.3352	0.0381	*
40 °C	2	Plastic vs. Alginate Film	0.1433	-0.005017 to 0.2917	0.0535	ns
40 °C	2	Plastic vs. Alginate Film + Vit. E	0.02333	-0.09840 to 0.1451	0.828	ns
40 °C	2	Plastic vs. Bioplastic	0.02667	-0.09506 to 0.1484	0.7737	ns
40 °C	3	Plastic vs. Alginate Film	-0.09	-0.2082 to 0.02816	0.1029	ns
40 °C	3	Plastic vs. Alginate Film + Vit. E	0.18	0.05983 to 0.3002	0.0133	*
40 °C	3	Plastic vs. Bioplastic	0.2067	0.05619 to 0.3571	0.0181	*
40 °C	5	Plastic vs. Alginate Film	0.1867	-0.06535 to 0.4387	0.1086	ns
40 °C	5	Plastic vs. Alginate Film + Vit. E	0.03333	-0.3737 to 0.4404	0.9567	ns
40 °C	5	Plastic vs. Bioplastic	0.1867	0.03867 to 0.3347	0.0236	*
40 °C	6	Plastic vs. Alginate Film	-0.08333	-0.2381 to 0.07148	0.2057	ns
40 °C	6	Plastic vs. Alginate Film + Vit. E	-0.3067	-0.3888 to -0.2246	0.0004	***
40 °C	6	Plastic vs. Bioplastic	0.1833	0.1111 to 0.2555	0.0021	**
70 °C	0	Plastic vs. Alginate Film	0	-0.1194 to 0.1194	>0.9999	ns
70 °C	0	Plastic vs. Alginate Film + Vit. E	0	-0.1194 to 0.1194	>0.9999	ns
70 °C	0	Plastic vs. Bioplastic	0	-0.1194 to 0.1194	>0.9999	ns
70 °C	1	Plastic vs. Alginate Film	0.2633	0.1748 to 0.3519	0.0011	**
70 °C	1	Plastic vs. Alginate Film + Vit. E	0.38	0.3018 to 0.4582	0.0002	***
70 °C	1	Plastic vs. Bioplastic	-0.44	-0.6117 to -0.2683	0.0043	**
70 °C	2	Plastic vs. Alginate Film	0.1067	0.03941 to 0.1739	0.0055	**
70 °C	2	Plastic vs. Alginate Film + Vit. E	0.3067	0.2021 to 0.4112	0.0003	***
70 °C	2	Plastic vs. Bioplastic	-0.1467	-0.2352 to -0.05815	0.0043	**
70 °C	3	Plastic vs. Alginate Film	0.04667	-0.04621 to 0.1395	0.3705	ns
70 °C	3	Plastic vs. Alginate Film + Vit. E	0.08667	-0.01619 to 0.1895	0.1147	ns
70 °C	3	Plastic vs. Bioplastic	-0.18	-0.2903 to -0.06967	0.0049	**
70 °C	5	Plastic vs. Alginate Film	0.2267	0.1097 to 0.3437	0.0015	**
70 °C	5	Plastic vs. Alginate Film + Vit. E	0.3	0.1785 to 0.4215	0.0004	***
70 °C	5	Plastic vs. Bioplastic	-0.23	-0.3238 to -0.1362	0.0007	***
70 °C	6	Plastic vs. Alginate Film	0.4833	0.3938 to 0.5728	<0.0001	****
70 °C	6	Plastic vs. Alginate Film + Vit. E	0.7333	0.6391 to 0.8276	<0.0001	****
70 °C	6	Plastic vs. Bioplastic	-0.26	-0.3460 to -0.1740	<0.0001	****

Statistical analysis was performed using two-way ANOVA considering storage time and packaging as factors, followed by Dunnett's post hoc test for comparisons against plastic packaging. Only selected comparisons relevant to the discussion are reported.

Table S8. Dunnett's multiple comparisons test (vs plastic packaging) for Total Antioxidant Capacity (%TAC) at each storage time. Two-way ANOVA was performed considering *storage time* and *packaging* as factors; post hoc comparisons were carried out using Dunnett's test vs plastic at the same storage time. Data are reported as adjusted p-values ($\alpha = 0.05$).

Drying temp	Storage time (months)	Comparison	Mean diff	95% CI of diff	Adjusted p value	Sig
40 °C	0	Plastic vs. Alginate Film	0	-1.705 to 1.705	>0.9999	ns
40 °C	0	Plastic vs. Alginate Film + Vit. E	0	-1.705 to 1.705	>0.9999	ns
40 °C	0	Plastic vs. Bioplastic	0	-1.705 to 1.705	>0.9999	ns
40 °C	1	Plastic vs. Alginate Film	-0.3333	-3.059 to 2.392	0.9205	ns

40 °C	1	Plastic vs. Alginate Film + Vit. E	3.333	0.6079 to 6.059	0.0284	*
40 °C	1	Plastic vs. Bioplastic	7.333	4.608 to 10.06	0.0026	**
40 °C	2	Plastic vs. Alginate Film	0.3333	-2.392 to 3.059	0.9205	ns
40 °C	2	Plastic vs. Alginate Film + Vit. E	2.667	-0.05835 to 5.392	0.063	ns
40 °C	2	Plastic vs. Bioplastic	4.333	1.608 to 7.059	0.0107	*
40 °C	3	Plastic vs. Alginate Film	-0.3333	-3.059 to 2.392	0.9205	ns
40 °C	3	Plastic vs. Alginate Film + Vit. E	4	1.274 to 6.726	0.0146	*
40 °C	3	Plastic vs. Bioplastic	3.667	0.9412 to 6.392	0.0223	*
40 °C	5	Plastic vs. Alginate Film	1.333	-1.392 to 4.059	0.4029	ns
40 °C	5	Plastic vs. Alginate Film + Vit. E	3.333	0.6079 to 6.059	0.0284	*
40 °C	5	Plastic vs. Bioplastic	2.333	-0.3921 to 5.059	0.1077	ns
40 °C	6	Plastic vs. Alginate Film	0.3333	-2.392 to 3.059	0.9205	ns
40 °C	6	Plastic vs. Alginate Film + Vit. E	2.667	-0.05835 to 5.392	0.063	ns
40 °C	6	Plastic vs. Bioplastic	1.667	-1.058 to 4.392	0.3198	ns
70 °C	0	Plastic vs. Alginate Film	0	-1.705 to 1.705	>0.9999	ns
70 °C	0	Plastic vs. Alginate Film + Vit. E	0	-1.705 to 1.705	>0.9999	ns
70 °C	0	Plastic vs. Bioplastic	0	-1.705 to 1.705	>0.9999	ns
70 °C	1	Plastic vs. Alginate Film	1.333	-0.3721 to 3.039	0.1034	ns
70 °C	1	Plastic vs. Alginate Film + Vit. E	4.333	2.628 to 6.039	0.001	**
70 °C	1	Plastic vs. Bioplastic	-6.333	-9.059 to -3.608	0.0041	**
70 °C	2	Plastic vs. Alginate Film	2	0.2946 to 3.705	0.0278	*
70 °C	2	Plastic vs. Alginate Film + Vit. E	4.333	2.628 to 6.039	0.001	**
70 °C	2	Plastic vs. Bioplastic	-4.333	-7.059 to -1.608	0.0107	*
70 °C	3	Plastic vs. Alginate Film	2	0.2946 to 3.705	0.0278	*
70 °C	3	Plastic vs. Alginate Film + Vit. E	4	2.295 to 5.705	0.0022	**
70 °C	3	Plastic vs. Bioplastic	-4.667	-7.392 to -1.941	0.0072	**
70 °C	5	Plastic vs. Alginate Film	2.667	0.9612 to 4.372	0.0127	*
70 °C	5	Plastic vs. Alginate Film + Vit. E	4.333	2.628 to 6.039	0.001	**
70 °C	5	Plastic vs. Bioplastic	-3.667	-6.392 to -0.9412	0.0223	*
70 °C	6	Plastic vs. Alginate Film	3.333	1.628 to 5.039	0.004	**
70 °C	6	Plastic vs. Alginate Film + Vit. E	5	3.295 to 6.705	0.0007	***
70 °C	6	Plastic vs. Bioplastic	-2.667	-5.392 to 0.05835	0.063	ns

Statistical analysis was performed using two-way ANOVA considering storage time and packaging as factors, followed by Dunnett’s post hoc test for comparisons against plastic packaging. Only selected comparisons relevant to the discussion are reported.

Two-way ANOVA for conventional packaging (plastic vs bioplastic).

To specifically assess differences between conventional packaging materials, a two-way mixed-effects ANOVA (REML) was applied considering storage time and packaging type (plastic vs bioplastic) as fixed factors. This analysis was performed separately for β-carotene (Table S9) and lycopene (Table S11) contents. When a significant interaction between storage time and packaging was detected, post hoc comparisons were carried out using Dunnett’s multiple comparisons test, with plastic packaging taken as the reference at the same storage time (Table S10 for β-carotene; Table

S11 for lycopene). Adjusted p-values were used to account for multiple comparisons ($\alpha = 0.05$). This approach allowed the identification of packaging-dependent differences at specific storage times, while avoiding overinterpretation of main effects when interaction terms were significant.

Table S9. Effect of drying temperature on β-carotene content (ppm) in tomato by-product powders stored in conventional packaging.

A mixed-effects two-way ANOVA (REML) was performed considering storage time and sample group (TBP-70 plastic, TBP-70 bioplastic, TBP-40 plastic, TBP-40 bioplastic) as factors. Post hoc comparisons were carried out using Dunnett's test, with TBP-70 plastic taken as reference at the same storage time. Adjusted p-values are reported ($\alpha = 0.05$).

Storage time (months)	Comparison (vs TBP-70 Plastic)	Mean diff (ppm)	95% CI of diff	Adjusted p value	Sig
2	TBP-70 Bioplastic	−0.463	−1.573 to 0.646	0.3003	ns
2	TBP-40 Plastic	0.093	−1.093 to 1.280	0.9601	ns
2	TBP-40 Bioplastic	−0.130	−1.142 to 0.882	0.8824	ns
6	TBP-70 Bioplastic	−0.700	−1.292 to −0.108	0.0290	*
6	TBP-40 Plastic	−0.147	−0.956 to 0.662	0.8263	ns
6	TBP-40 Bioplastic	−0.247	−0.937 to 0.443	0.4899	ns
8	TBP-70 Bioplastic	−0.230	−1.692 to 1.232	0.8099	ns
8	TBP-40 Plastic	−0.290	−1.099 to 0.519	0.4651	ns
8	TBP-40 Bioplastic	0.010	−0.581 to 0.601	0.9999	ns
11	TBP-70 Bioplastic	−0.260	−0.555 to 0.035	0.0738	ns
11	TBP-40 Plastic	−0.340	−0.635 to −0.045	0.0318	*
11	TBP-40 Bioplastic	−0.083	−0.358 to 0.191	0.5932	ns

Table S10. Dunnett's multiple comparisons test (vs plastic packaging) for β-carotene content (ppm) at each storage time.

Two-way ANOVA was performed considering storage time and packaging as factors for TBP-40 samples. Post hoc comparisons were carried out using Dunnett's test vs plastic packaging at the same storage time. Data are reported as mean differences, 95% confidence intervals (CI) and adjusted p-values ($\alpha = 0.05$).

Drying temp	Storage time (months)	Comparison	Mean diff (ppm)	95% CI of diff	Adjusted p value	Sig
40 °C	2	Plastic vs Bioplastic	−0.223	−1.344 to 0.897	0.814	ns
40 °C	2	Plastic vs Alginate Film	0.450	−0.732 to 1.632	0.462	ns
40 °C	2	Plastic vs Alginate Film + Antioxidant	0.520	−0.662 to 1.702	0.371	ns
40 °C	6	Plastic vs Bioplastic	−0.100	−0.931 to 0.731	0.942	ns
40 °C	6	Plastic vs Alginate Film	0.443	−0.366 to 1.253	0.229	ns
40 °C	6	Plastic vs Alginate Film + Antioxidant	0.470	−0.442 to 1.382	0.197	ns
40 °C	8	Plastic vs Bioplastic	−0.183	−0.999 to 0.633	>0.05	ns
40 °C	8	Plastic vs Alginate Film	0.390	−0.501 to 1.281	>0.05	ns
40 °C	8	Plastic vs Alginate Film + Antioxidant	0.410	−0.498 to 1.318	>0.05	ns
40 °C	11	Plastic vs Bioplastic	−0.260	−1.104 to 0.584	>0.05	ns
40 °C	11	Plastic vs Alginate Film	0.360	−0.590 to 1.310	>0.05	ns
40 °C	11	Plastic vs Alginate Film + Antioxidant	0.380	−0.575 to 1.335	>0.05	ns

Table S11. Effect of conventional packaging on lycopene content (ppm) in tomato by-product powders dried at different temperatures.

A mixed-effects two-way ANOVA (REML) was performed considering storage time and sample group (TBP-70 plastic, TBP-70 bioplastic, TBP-40 plastic, TBP-40 bioplastic) as factors. Post hoc comparisons were carried out using Dunnett's test, with TBP-70 plastic taken as reference at the same storage time. Adjusted p-values are reported ($\alpha = 0.05$).

Storage time (months)	Comparison (vs TBP-70 Plastic)	Mean diff (ppm)	95% CI of diff	Adjusted p value	Sig
2	TBP-70 Bioplastic	−0.567	−0.848 to −0.285	0.0045	**

2	TBP-40 Plastic	0.063	−0.216 to 0.343	0.6805	ns
2	TBP-40 Bioplastic	0.350	0.049 to 0.651	0.0354	*
6	TBP-70 Bioplastic	−0.160	−0.751 to 0.431	0.6712	ns
6	TBP-40 Plastic	−0.100	−0.658 to 0.458	0.7907	ns
6	TBP-40 Bioplastic	−0.080	−0.629 to 0.469	0.8745	ns
8	TBP-70 Bioplastic	−0.070	−0.365 to 0.225	0.7412	ns
8	TBP-40 Plastic	−0.130	−0.425 to 0.165	0.3706	ns
8	TBP-40 Bioplastic	−0.010	−0.293 to 0.273	0.9982	ns
11	TBP-70 Bioplastic	−0.030	−0.325 to 0.265	0.9645	ns
11	TBP-40 Plastic	−0.030	−0.325 to 0.265	0.9645	ns
11	TBP-40 Bioplastic	0.000	−0.274 to 0.274	>0.9999	ns

Table S12. Effect of innovative packaging on lycopene content (ppm) in tomato by-product powders dried at 40 °C (TBP-40).

A mixed-effects two-way ANOVA (REML) was performed considering storage time and packaging (plastic, bioplastic, alginate film, alginate film + antioxidant) as factors. Post hoc comparisons were carried out using Dunnett’s test, with plastic packaging taken as reference at the same storage time. Adjusted p-values are reported ($\alpha = 0.05$).

**Storage time (months)**	**Comparison (vs Plastic)**	**Mean diff (ppm)**	**95% CI of diff**	**Adjusted p value**	**Sig**
2	Bioplastic	0.2867	0.1517 to 0.4217	0.0044	*
2	Alginate Film	−0.0200	−0.4395 to 0.3995	0.9894	ns
2	Alginate Film + Antioxidant	−0.0200	−0.3995 to 0.3595	0.9894	ns
6	Bioplastic	0.0200	−0.2919 to 0.3319	0.9900	ns
6	Alginate Film	−0.0900	−0.2054 to 0.0254	0.6025	ns

6	Alginate Film + Antioxidant	−0.1000	−0.2314 to 0.0314	0.6002	ns
8	Bioplastic	0.1200	−0.1625 to 0.4025	0.3903	ns
8	Alginate Film	−0.1300	−0.4554 to 0.1954	0.3706	ns
8	Alginate Film + Antioxidant	0.1500	−0.1454 to 0.4454	0.2865	ns
11	Bioplastic	0.0300	−0.2438 to 0.3038	0.9532	ns
11	Alginate Film	−0.0200	−0.2754 to 0.2354	0.9885	ns
11	Alginate Film + Antioxidant	0.0300	−0.2654 to 0.3254	0.9645	ns

Table S13. Oxygen Transmission Rate (OTR) and Water Vapor Transmission Rate (WVTR) of conventional plastic and bioplastic packaging materials measured at the beginning of storage (T0) and after 11 months of storage (T11). OTR was determined according to ASTM D3985, while WVTR was measured following ASTM F1249. Values are reported as mean ± SD (n = 3).

**Sample**	**Timepoint**	**OTR (cc / m^2 day)**	**WVTR (g / m^2 day)**
**Plastic**	T0	17.12 ± 1.45	8.84 ± 1.32
**Plastic**	T11	24.47 ± 1.84	10.05 ± 1.12
**Bioplastic**	T0	741 ± 4.45	125.6 ± 2.64
**Bioplastic**	T11	1407 ± 5.96	203.56 ± 3.07

*Alginate-based films. with or without antioxidant addition. were not included in OTR and WVTR measurements due to their highly permeable nature. which prevented reliable determination of transmission rates using standard ASTM methods.
